# High-Frequency TRNS Reduces BOLD Activity during Visuomotor Learning

**DOI:** 10.1371/journal.pone.0059669

**Published:** 2013-03-20

**Authors:** Catarina Saiote, Rafael Polanía, Konstantin Rosenberger, Walter Paulus, Andrea Antal

**Affiliations:** 1 Department of Clinical Neurophysiology, Georg-August University of Göttingen, Göttingen, Germany; 2 Laboratory for Social and Neural Systems Research, Department of Economics, University of Zurich, Zurich, Switzerland; University of Bologna, Italy

## Abstract

Transcranial direct current stimulation (tDCS) and transcranial random noise stimulation (tRNS) consist in the application of electrical current of small intensity through the scalp, able to modulate perceptual and motor learning, probably by changing brain excitability. We investigated the effects of these transcranial electrical stimulation techniques in the early and later stages of visuomotor learning, as well as associated brain activity changes using functional magnetic resonance imaging (fMRI). We applied anodal and cathodal tDCS, low-frequency and high-frequency tRNS (lf-tRNS, 0.1–100 Hz; hf-tRNS 101–640 Hz, respectively) and sham stimulation over the primary motor cortex (M1) during the first 10 minutes of a visuomotor learning paradigm and measured performance changes for 20 minutes after stimulation ceased. Functional imaging scans were acquired throughout the whole experiment. Cathodal tDCS and hf-tRNS showed a tendency to improve and lf-tRNS to hinder early learning during stimulation, an effect that remained for 20 minutes after cessation of stimulation in the late learning phase. Motor learning-related activity decreased in several regions as reported previously, however, there was no significant modulation of brain activity by tDCS. In opposition to this, hf-tRNS was associated with reduced motor task-related-activity bilaterally in the frontal cortex and precuneous, probably due to interaction with ongoing neuronal oscillations. This result highlights the potential of lf-tRNS and hf-tRNS to differentially modulate visuomotor learning and advances our knowledge on neuroplasticity induction approaches combined with functional imaging methods.

## Introduction

The acquisition of a new motor skill follows a time-course that can be divided in two stages. The early stage is characterized by a marked improvement in performance in a relatively short period of time, followed by further progress in a gradual manner. This process is reflected in the dynamics of brain activity which can be observed regionally and as connectivity changes using fMRI [1]–[5]. A decrease in activity of primary sensorimotor cortex, supplementary motor area (SMA), anterior cingulate cortex (ACC), caudate and posterior parietal and frontal regions has been observed during early learning, whereas the putamen, thalamus and cerebellar dentate showed the opposite behaviour [1]. Instead, long term motor learning has been associated with increased activity in the sensorimotor cortex and striatum [2].

Transcranial direct current stimulation (tDCS) is a technique that allows non-invasive modulation of brain excitability through the application of weak electrical current (most commonly using 1 mA intensity). Generally, the application of 10 minutes anodal tDCS over the primary motor cortex (M1) is followed by an increase and cathodal by a decrease in excitability, which can last for up to 90 min [6], [7]. In addition to the modulation of cortical excitability, it has been shown that the effects of tDCS have functional repercussions at a perceptual [8], [9], cognitive [10]–[12] and motor level [9], [13], [14].

Transcranial random noise stimulation (tRNS) is a form of alternating current stimulation (tACS) applied at random frequencies between 0.1 and 640 Hz, which can lead to an increase in performance of implicit motor or perceptual learning tasks [15]–[17]. Its effects on cortical excitability have also been shown to depend on the frequency range used for stimulation: high-frequency tRNS (hf-tRNS; 101–640 Hz) increases cortical excitability whereas low-frequency tRNS (lf-tRNS; 0.1–100 Hz) does not induce significant alterations [16].

The M1 has been recognized as a major structure in motor sequence learning and has therefore been a target brain region in several studies employing transcranial stimulation in an attempt to characterize its role in motor learning. Indeed, anodal tDCS over the M1 during early learning improved performance of a serial reaction time task (SRTT) [12] and a visuomotor task [18]. However, when tDCS was applied after the initial stage of learning, after achieving stable performance, anodal tDCS had no effect and cathodal tDCS improved performance of the visuomotor task [15]. On the other hand, stimulating before learning improved performance regardless of tDCS polarity applied over the M1 [19].

The application of tDCS as well as tRNS over the M1 has been suggested to modulate functional networks involving motor cortical and subcortical structures during rest and simple motor tasks [20]–[24]. In line with these hypotheses, a previous functional magnetic resonance imaging (fMRI) study reported increased activity during a SRTT in ipsilateral SMA and M1 following anodal and contralateral M1 and dorsal premotor cortex after cathodal tDCS [25].

This study aimed at investigating how external modulation of excitability of the M1 influences the dynamics of visuomotor learning and corresponding functional networks during two stages of learning. We applied tRNS and tDCS during performance of a visuomotor tracking task, while measuring BOLD activity changes. The task used here was adapted from previous studies and consisted in learning a pattern of variable hand pressure movements with visual feedback [1].

According to previous results, we expected an improvement in performance during the first stage of learning due to anodal tDCS and a similar or even greater increase in performance by high-frequency stimulation, considering previous electrophysiological results [16]. We were interested in observing whether the difference between groups would be observable and maintained after stabilization of performance. Furthermore, we hypothesised that hf-tRNS would be responsible for a greater improvement of performance than lf-tRNS in parallel with the previously observed effects regarding brain excitability [16], [17].

## Methods

### Subjects

A group of 52 healthy volunteers took part in the study (22 male, mean age: 27±6 years, age range: 20–50 years, right-handedness assessed by self-report). The participants fulfilled the following criteria: no history of neurological or psychiatric disorders, no drug abuse, no alcoholism, normal or corrected to normal visual acuity and no metal implants. Two subjects were unable to learn the task and were excluded from further analysis. Fifty subjects remained, 10 in each stimulation condition: anodal tDCS (3 male, mean age: 28±8 years, age range: 22–50 years), cathodal tDCS (6 male, mean age: 25±4, age range: 20–32 years), sham (2 male, mean age: 28±7, age range: 23–44 years), high-frequency tRNS (4 male, mean age: 27±3, age range: 20–27 years) and low-frequency tRNS (7 male, mean age: 31±7, age range: 24–37 years). All participants gave written informed consent. The experiments conform to the Declaration of Helsinki, and the experimental protocol was approved by the Ethics Committee of the University of Göttingen.

### Visual Stimuli and Experimental Design

Stimuli were presented using Presentation (version 14.9, Neurobehavioral Systems, Albany, NY). Subjects viewed the stimuli through a set of MR-compatible LCD goggles (Resonance Technology, Northridge, USA), covering a visual field of 20° and 30° in the vertical and horizontal direction, respectively. Visual stimuli consisted of two columns on a light green (RGB code: R = 155, G = 206, B = 155) background positioned in equal distances from the midline of the goggles-LCD. During the task periods, the height-level of the sample column (left) varied at a constant speed. The height-level of the right column was controlled by the subject according to the pressure applied on a custom-made air-filled rubber ball held with the right hand. The subjects were asked to mimic the sample column's height changes as well as possible. The ball was connected to a sensor, which converted pressure changes into digital signals with adjustable gain. Subjects received feedback on their performance through the colour of the column they controlled: green if the pixel difference between columns was below 40 pixels, yellow if the difference was between 40 and 100 pixels and red whenever the difference exceeded 100 pixels. Throughout the task, information regarding the sample and subject's column height-level was saved in a text file with sampling frequency of 20 Hz. Before the beginning of each run, subjects were asked to press the ball as hard as possible in order to calibrate the digital sensor gain according to each subject’s strength.

**Figure 1:**
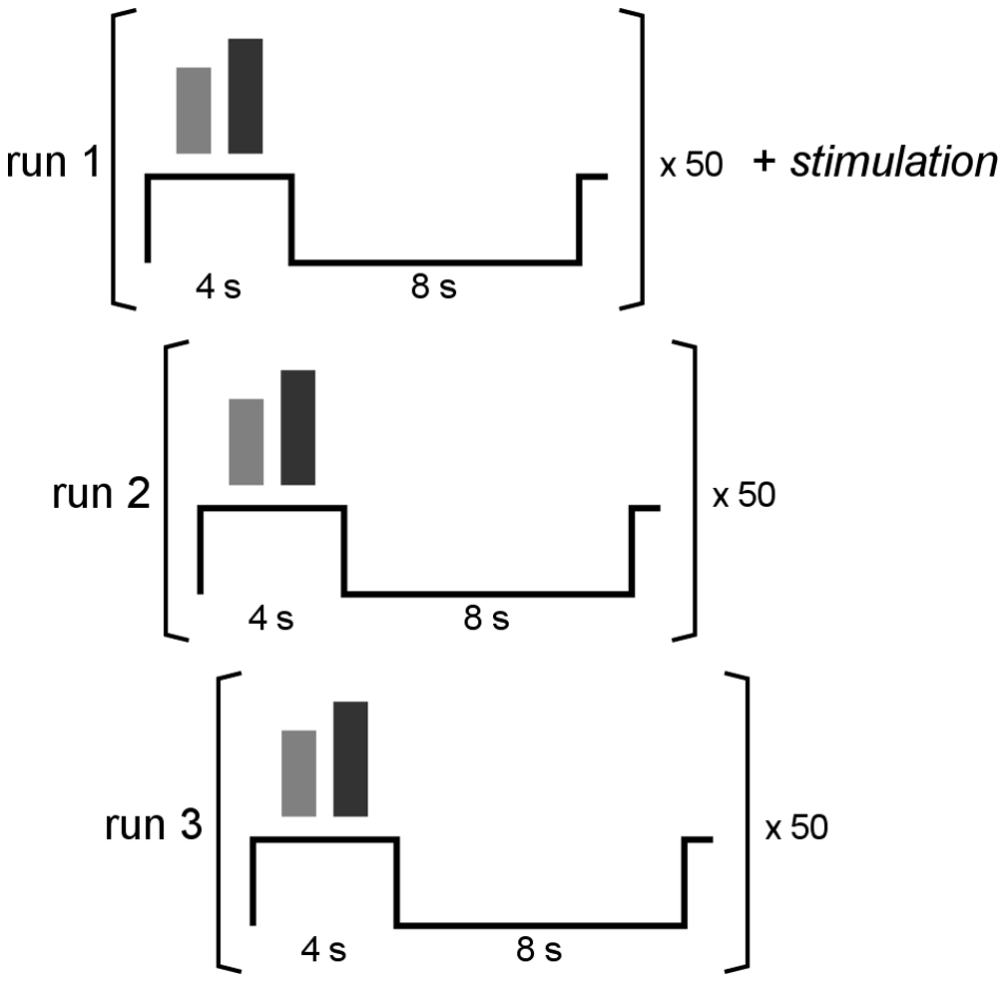
Experimental paradigm. In each block the task had a duration of 4 s followed by 8 s rest. There were 50 repetitions of the block in one run. The experiment was completed after 3 runs and stimulation was delivered during the first run.

The experiment followed a block design (Figure 1). In each trial, the task period lasted 4 seconds and the pause period lasted 8 seconds, during which the participants saw a fixation cross. The complete experiment consisted of three runs, each with 50 trials (Figure 1).

### Transcranial Electrical Stimulation

Stimulation was delivered using a pair of rubber electrodes of 5×7 cm connected to a battery-driven stimulator (Version DC-Stimulator-Plus, NeuroConn GmbH, Ilmenau, Germany) in a previously described setup compatible with MR environment [21].

Stimulation was applied during the first run and lasted for 10 minutes, at an intensity of 1 mA, with 20 seconds fade in and 10 seconds fade out in order to minimize sensory perception. In the anodal tDCS group, the positive electrode was placed over the left M1 and the reference over the contra-lateral orbita and the opposite for the cathodal tDCS group. In the sham group, stimulation consisted solely of 20 seconds fade in and 10 seconds fade out. With regard to tRNS, the frequency spectrum was divided into two ranges: low-frequency (0.1–100 Hz) and high-frequency (101–640 Hz). The subjects were blinded with regard to the type of stimulation.

### Imaging Data Acquisition

Imaging data was acquired at 3T (Magnetom TIM Trio, Siemens Healthcare, Erlangen, Germany) using a standard eight-channel phased array head coil. Subjects were placed supine inside the magnet bore and wore headphones for noise protection. Vital functions were monitored throughout the experiment. At the beginning of each session, T1-weighted structural images were obtained using a 3D turbo fast low angle shot (FLASH) MRI sequence with 1 mm3 isotropic resolution (repetition time (TR) = 1950 ms, inversion time = 1100 ms, echo time (TE) = 3.93 ms, flip angle = 12°). For BOLD functional images a multislice T2*-sensitive echo-planar imaging (EPI) sequence (TR = 2000 ms, TE = 36 ms, flip angle = 70° was used at a resolution of 2×2 mm2. Twenty-two consecutive 4 mm-thick slices angulated in an axial-to-coronal orientation, covering the brain areas of interest (M1, SMA, occipital lobe, basal ganglia and cerebellum) were acquired. Previous studies have provided evidence that simultaneous tDCS and EPI does not cause heating under the electrodes [26] and does not have a detrimental effect on the quality of the data: maximal detected signal-to-noise reduction was of 8% and no artifacts were detected in brain tissue [21], [26]. Voxelwise statistics on the mean EPI images showed no systematic effect of stimulation on the mean BOLD signal (Figure S1).

### Analysis of Behavioural Data

Performance in each trial was measured as the difference between the required and the applied pressure (tracking error). After removing the first trial of every run, the tracking error was averaged for each 5 consecutive trials (block) and normalized with respect to the second trial. Improvement in performance was determined by the ratio of current with initial tracking error. A 30 (block)×5 (anodal, cathodal, high-frequency, low-frequency and sham stimulation) repeated measures analysis of variance (ANOVA) was applied using IBM SPSS Statistics, version 20.

Additionally, we averaged tracking errors across each run and did a 3 (run)×5 (stimulation condition) repeated measures ANOVA, in order to better understand the effects of stimulation and increase signal-to-noise ratio of the behavioural data.

### Analysis of Imaging Data

The analysis of fMRI data was carried out using FEAT (FMRI Expert Analysis Tool) Version 5.98, part of FSL (FMRIB's Software Library, www.fmrib.ox.ac.uk/fsl). Pre-processing of functional datasets included the following steps: motion correction [27]; non-brain removal [28]; slice timing correction, spatial smoothing (Gaussian kernel, 8 mm FWHM); mean-based intensity normalization of all volumes by the same factor; and high-pass temporal filtering (Gaussian-weighted least squares straight line fitting, 15 s cut-off). Each subject’s functional datasets were registered to the T1-weighted structural image and to the MNI152 standard template using FLIRT [27], [29]. The time-series of each dataset was analysed using a General Linear Model (GLM) approach with autocorrelation correction [30]. The hemodynamic response function (HRF) was modelled as a Gamma variate (phase = 0; standard deviation = 3s, mean lag = 6s).

For the first-level analysis, one explanatory variable (EV) was defined as a square function representing the on-off periods of the task to model motor-related activity (Mov-Rest) and a second EV was defined using the behavioural scores of each participant, orthogonalized with the first EV to model performance-related activity. The temporal derivatives were also included in the model. Additionally, the six motion parameters calculated during head motion correction were added as covariates of no interest to remove potential signal variability caused by non-corrected motion.

A second-level fixed-effects analysis was performed on each subject’s 3 datasets for individual averaging of activation. These were afterwards used in a higher-level analysis to calculate global average activity, for each of the first-level contrasts.

The results from the second-level analysis were entered into a third-level mixed-effects analysis modelling an ANOVA to detect regions with an effect of stimulation. Pairwise contrasts of stimulation conditions were then defined to determine specific changes according to the type of stimulation. Given that there are 4 stimulation conditions, an ANOVA including all the groups will not be sensitive enough to detect changes, if they happen in only one of the groups. However, being an exploratory study, the main goal was to determine whether any stimulation condition is able to modulate the task performance. Thus, we performed 2 separate ANOVAs for tDCS and tRNS. The probability Z-maps were thresholded with clusters determined by Z >2.3 and a significance threshold P = 0.05 with (cluster) correction for multiple comparisons, for all analysis described above.

Since the type of stimulation is a between-subjects factor and the run is a within-subjects factor, the effect of run and interaction between run and stimulation condition were calculated separately by entering each first-level analysis into a repeated measured ANOVA regressing out each subject’s average activity. Each pair of runs (*run1-run2*, *run1-run3*, *run2-run3*) was then contrasted to evaluate activity changes related to motor learning in each step of the experiment. Statistical images were thresholded with clusters determined by Z >2.3 and a significance threshold P = 0.05, cluster corrected.

## Results

### Behavioural Results

**Figure 2:**
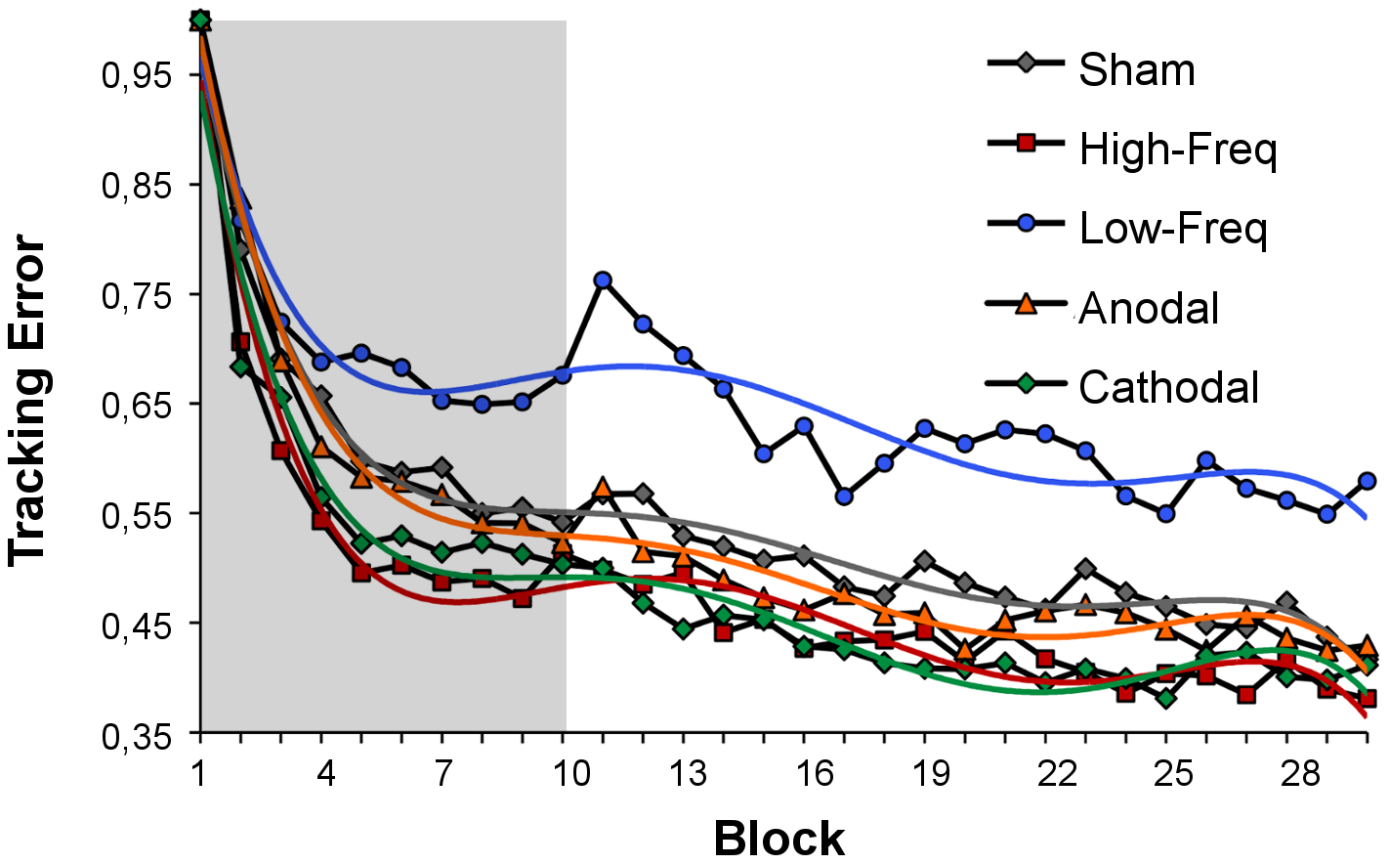
Changes in tracking error relative to the first trial. Shaded area corresponds to the stimulation period. Fifth Polynomial trendlines are superimposed on the data for easier visualization.

Fifty subjects out of 52 were able to learn the task reducing the tracking error by approximately 50% (53±13%) until the end of the experiment (Figure 2). Performance increased by 40% (41±14%) in the first run, when learning was more pronounced, while it progressed slowly through the rest of the experiment (Figure 2). A significant effect of block was found for the global analysis (F = 88,322; df = 29; p<0,001). Results of pairwise comparisons are presented in Table S1. Although there was a tendency for higher tracking error with lf-tRNS and lower tracking error with cathodal tDCS and hf-tRNS, there was no significant effect of stimulation (F = 1,464; df = 4; p = 0,115) or interaction of block and stimulation (F = 0,641; df = 116; p = 0,999). Averaging the tracking error across each run resulted in again a significant effect of run (F = 101,776; p<0,001; df = 2) but still no significant results were found regarding stimulation condition (F = 1,128; p = 0,355; df = 4) or run and stimulation interaction (F = 0,391; p = 0,923; df = 8).

### Imaging Results

Global average of motor-related activity (Figure S2) revealed an extensive network of brain regions comprising the primary and premotor cortices, SMA, prefrontal cortex, occipital cortex, thalamus and basal ganglia (Table 1). Performance related activity (Figure S3) showed a similar network with the exception of the primary and premotor cortices (Table 2).

**Table 1:** Peak voxel intensity and coordinates for brain regions showing motor task-related activity.

		MNI Coordinates		
Anatomical Region	*Z*	*x*	*y*	*z*
L precentral gyrus	8,9	−58	6	22
R precentral gyrus	9,6	54	10	20
L postcentral gyrus	9,5	−4	2	6
L supplementary motor area	9,5	−4	2	46
R middle frontal gyrus	9,9	38	0	50
L prefrontal cortex	3,9	−36	38	4
R prefrontal cortex	6,8	38	42	22
anterior cingulate cortex	9,9	8	16	34
L temporal occipital fusiform cortex	9,5	−22	−78	−14
R temporal occipital fusiform cortex	9,9	22	−74	−18
L lateral occipital cortex (inf)	9,8	−44	−78	2
R lateral occipital cortex (inf)	10,1	48	−76	0
L occipital pole	10,2	−2	−90	16
R occipital pole	8,8	18	−94	18
L putamen	9,3	−22	8	2
R putamen	9,7	24	10	−2
R pallidum	8,9	16	6	0
L pallidum	8,7	−18	−2	−2
L thalamus	9,8	−14	−22	4
R thalamus	6,5	10	−20	6

**Table 2:** Peak voxel intensity and coordinates for brain regions showing performance-related activity.

		MNI Coordinates		
Anatomical Region	*Z*	*x*	*y*	*z*
L precentral gyrus	6,5	−44	0	42
R postcentral gyrus	5,7	54	−20	50
supplementary motor area	5,9	−2	8	56
L superior frontal gyrus	5,4	−4	34	42
R middle frontal gyrus	5,8	44	6	52
L middle frontal gyrus	6,4	−48	10	32
R inferior frontal gyrus	7,4	54	32	−6
L inferior frontal gyrus	6,4	−50	18	−4
L prefrontal cortex	4,8	−28	54	24
R prefrontal cortex	4,2	30	54	28
anterior cingulate cortex	5	4	20	28
L middle temporal gyrus	6,2	−62	−46	−4
R insula	4,4	36	−20	6
L temporal occipital fusiform cortex	6,9	−26	−52	−14
R occipital fusiform cortex	5,8	30	−84	−16
L lateral occipital cortex	6,7	−38	−86	22
R lateral occipital cortex	6,1	40	−76	32
Cuneous	6,6	0	−88	24
Precuneous	5,9	6	−50	52

**Figure 3:**
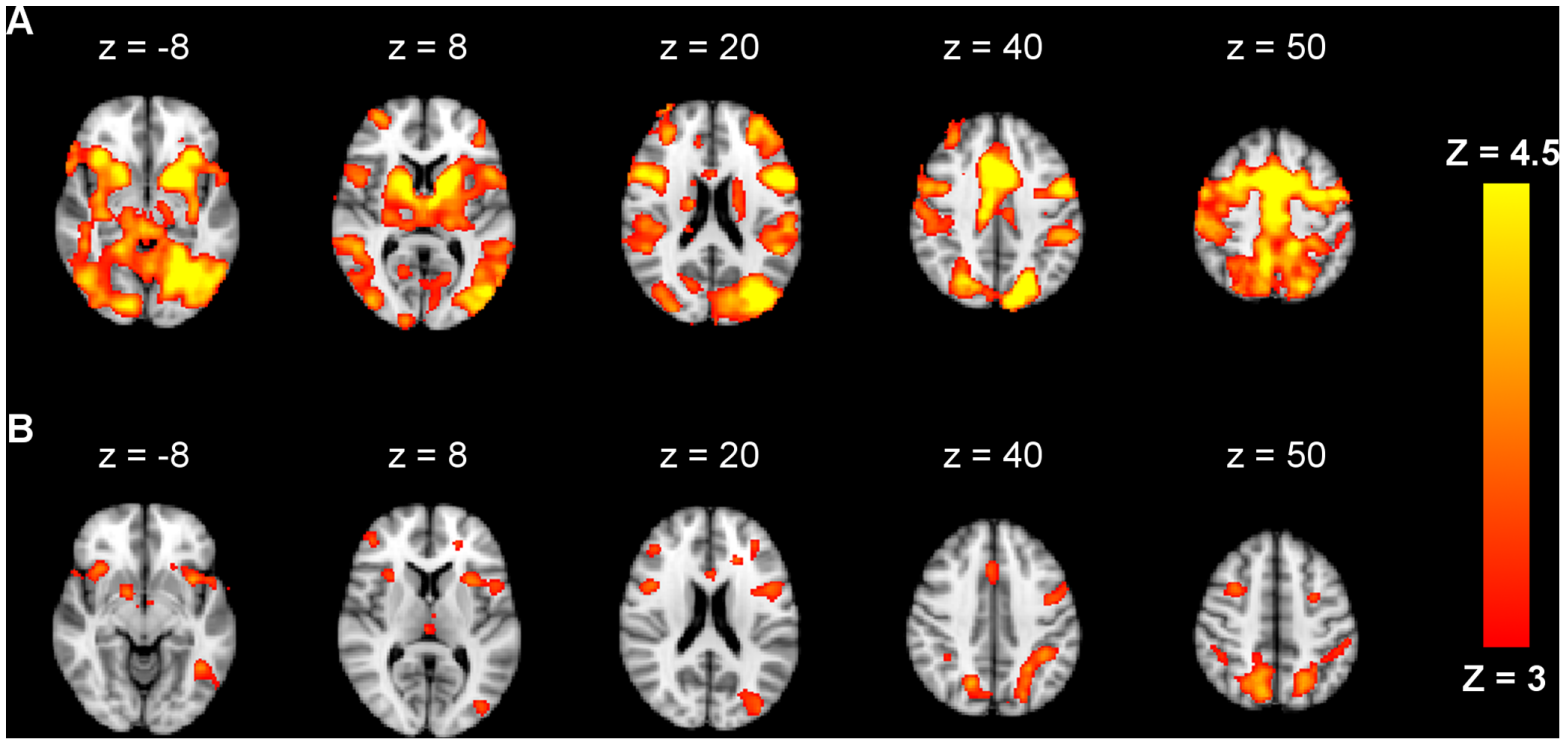
Motor task-related decrease of brain activity during and after stimulation. Activity decreased with time for contrasts *run1-run2* (A) in primary and premotor cortices, supplementary motor area (SMA), prefrontal cortex, occipital cortex, thalamus and basal ganglia and *run2-run3* (B) in the precuneous, superior parietal cortex, middle and inferior frontal gyrus, right prefrontal cortex, left inferior LOC and basal ganglia (Z>3, P<0.05, corrected).

**Figure 4:**
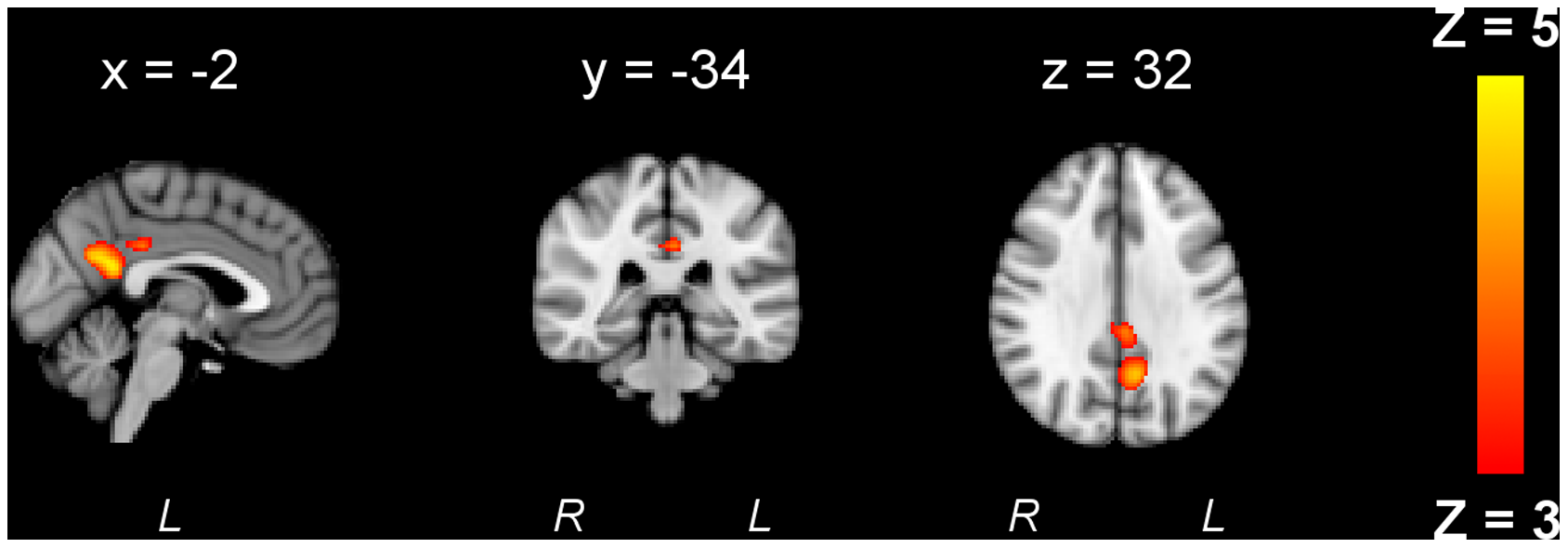
Motor task-related increase of brain activity during and after stimulation. Average of brain regions showing an increase in activity in the posterior cingulate cortex with time for contrast *run3-run1* (Z>3, P<0.05, corrected).

Activity decreased in the motor task-related network (Figure 3). Most of these changes occurred in the beginning of the experiment, as observed in the activity map of contrast *run1-run2* in the premotor cortex, M1, SMA, left LOC, left temporal occipital fusiform cortex and basal ganglia (Figures 3A). Activation further reduced from the second to the third run in areas comprising the precuneous, superior parietal cortex, middle and inferior frontal gyrus, right prefrontal cortex, left inferior lateral occipital cortex (LOC) and basal ganglia (Figure 3B). The posterior part of the cingulate gyrus, showed an increase in activity with time, for the contrast *run3-run1* (Figure 4). No activity was found for the contrast *run3-run2*.

**Figure 5:**
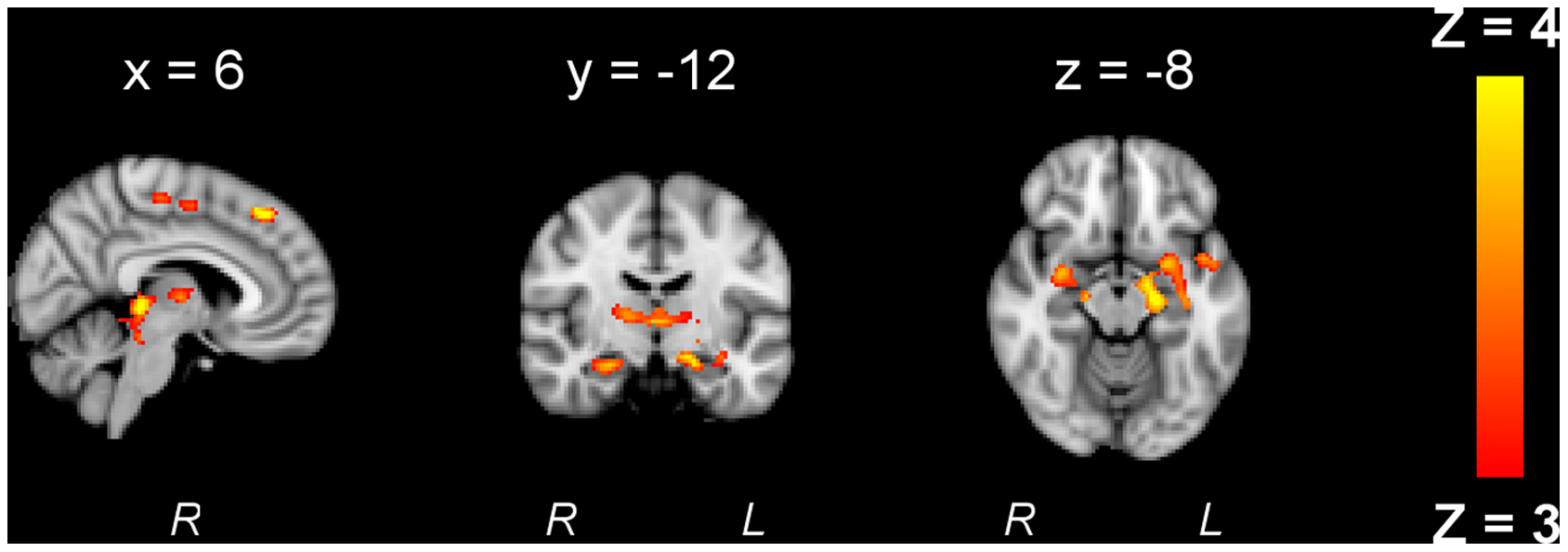
Performance related decrease of brain activity during and after stimulation. Activity decreased with time for contrast *run1-run2* in the paracingulate gyrus, superior frontal gyrus, thalamus and hippocampus. (Z>3, P<0.05, corrected).

Regarding performance-related activity, significant changes were detected bilaterally in the the paracingulate gyrus and superior frontal gyrus (x = −12, y = 22, z = 52), thalamus and hippocampus, where activity decreased from the first to the second run (Figure 5). No significant changes were found for contrasts *run2-run3* and no significant increase in brain activity was found.

### Stimulation-related Activity Changes

**Figure 6:**
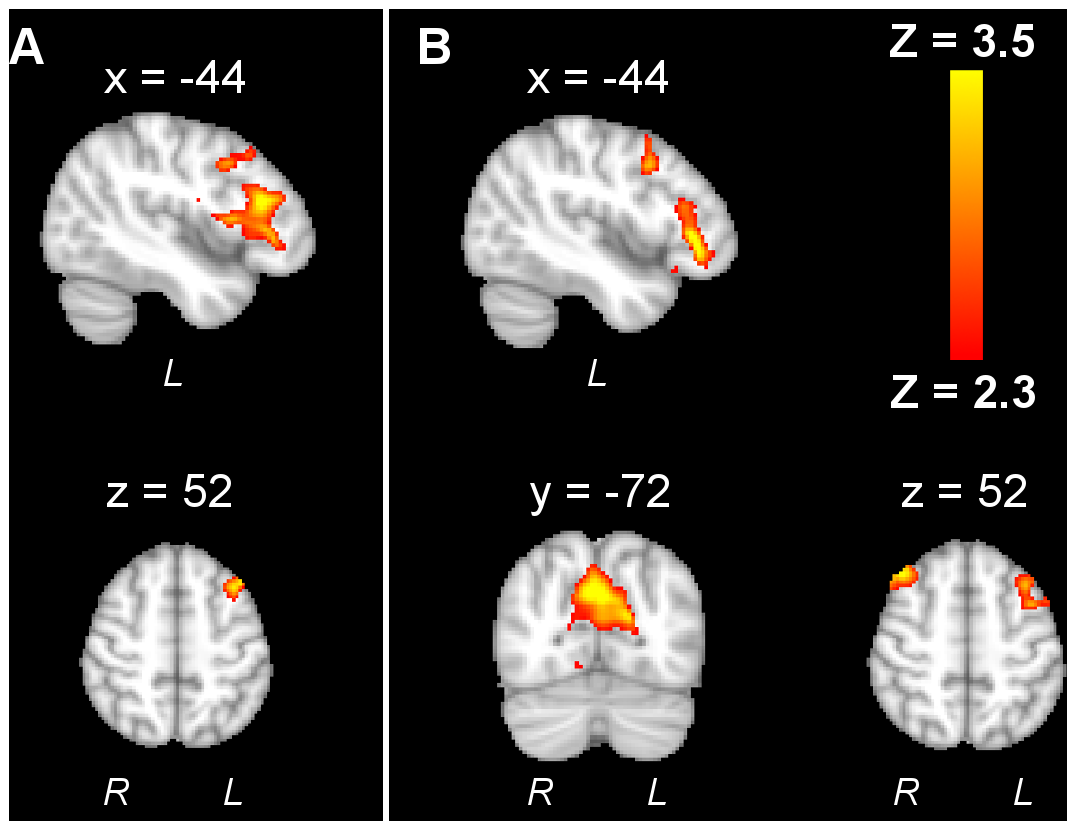
Regions of decreased activity for hf-tRNS. Contrast *sham- Hfreq* (A) revealed changes in the left frontal cortex. Contrast *Lfreq-Hfreq* (B) revealed additional changes in right frontal cortex and precuneous.

Regarding the different stimulation conditions, no significant effect of stimulation was detected with a global ANOVA. However, separate ANOVAs revealed a significant effect of tRNS for motor task-related activity but not for performance-related activity. The contrast *sham-Hfreq* revealed descreased motor task-related activity in the left frontal cortex (x = −44, y = 28, z = 18) when comparing hf-tRNS with sham (Figure 6A). The contrast *Lfreq-Hfreq* showed that compared to lf-tRNS, hf-tRNS was associated with decreased activity in the left frontal cortex (x = −44, y = 32, z = 12), precuneous (x = 4, y = −72, z = 32) and right frontal cortex (x = 38, y = 26, z = 52) (Figure 6B). No significant changes in motor-related or performance-related activity due to tDCS were found.

## Discussion

In this study, we investigated the effect of tDCS and tRNS on visuomotor learning using a task that requires subjects to learn a continuous pattern of pressure applied with right hand in response to a visual cue. We hypothesised that anodal tDCS and hf-tRNS would speed the learning process. However, on the behavioural level neither tDCS nor tRNS resulted in significant modulation of learning. Nevertheless, there was a tendency for cathodal tDCS and hf-tRNS to improve learning and for lf-tRNS to worsen it. When applied during a passive condition, hf-tRNS and cathodal tDCS lead to opposite changes in brain excitability: cathodal reduces and tRNS increases it [16]. However, when stimulating while subjects performed a motor task, compressing a rubber ball with the right hand, the effect of tRNS is inverted, and excitability decreases [16]. Thus, changes in excitability after a motor task are paralleled by the changes in learning that we found.

It has been described in several studies [5], [31] that learning a motor skill is a process that encompasses different stages, namely an early fast skill acquisition followed by slow gradual improvement. In agreement with previous work with the same task [1], [4], our results show that the fast learning stage occurred in the first 3 to 4 blocks (Table S1). In all stimulation conditions we found that motor task-related activity decreased with time in the M1, SMA, primary somatosensory cortex, premotor cortex, prefrontal cortex, frontal gyrus, insula, ACC and precuneous. In the ACC, activity reduced during learning, possibly due to an increase in movement automaticity [1], as this region is involved in effector functions and attention [32] . However, the cingulate cortex is known to be functional heterogeneous and the posterior portion has an evaluative role, being involved in spatial orientation and memory [32], which may explain the opposite change in activity. Performance-related activity decreased in the prefrontal cortex from the first to the second runs. as previously reported [1], in agreement with the behavioural results showing a stabilization of performance in the first run.

With regards to functional responses, a relative decrease in M1 activity was found in a previous study during finger-movement after whole spectrum tRNS comparing to sham [20]. In our case activity reduction was found instead in the visual cortex, precuneous and left pFC when comparing hf-tRNS with lf-tRNS and sham. This could be due to the task that was used, where an integration of visual and motor information is required for acquisition of skill, in comparison with a simple motor task such as finger-tapping. The precuneus is known to be involved in motor coordination as well as processing of visuospatial information and attentive tracking, being connected to prefrontal cortex, dorsal premotor cortex and SMA [33].

There are only two studies combining tRNS with a learning paradigm. Using a 2-back task, tRNS was found to be ineffective in modulating working memory [11]. It was recently documented that learning of a visual perception task was facilitated by hf-tRNS [17]. However, here tRNS was applied with 1.5 mA intensity over V1. Also, stimulation lasted for 22 minutes and the size of electrodes was markedly different, as the area of the active electrode was 16 cm^2^ and of the reference electrode 60 cm^2^. In particular, hf-tRNS significantly improved accuracy comparing to sham and anodal or cathodal tDCS, but not in comparison with lf-tRNS which showed a tendency to improve performance. In our study, the effect of lf-tRNS in learning was non-significant as well, but only a tendency to worsen learning. Also, regarding brain excitability, lf-tRNS had no effect on MEP amplitude during rest [16].

It has been suggested that tRNS modulates ongoing cortical oscillations [34], [35]. One potential mechanism is the summation of sub-threshold stimuli [17], thus depending on the time-constant of the neuron. This has been estimated to be between 1 and 10 ms [36], meaning that frequencies between 100 and 1000 Hz would be more effective for stimulation. However, different types of neurons have been shown to activate at different frequencies [37], possibly explaining the opposite effects of hf-tRNS and lf-tRNS. The influence of tRNS on neuronal synchronization provides a possible explanation for the effects of tRNS on BOLD activity: an increase in neuronal synchronization by hf-tRNS would lead to greater efficiency and a consequent decrease of activity [20].

We found no significant changes in BOLD activity due to tDCS. This was unexpected, taking into account previous studies of M1 stimulation [20]. However, it should be taken into account that the motor task in these studies consisted of simple finger-tapping, therefore it is not possible to compare performance related changes directly.

Our study has several limitations: (1) This work was an exploratory study, therefore our sample size might not have been large enough to detect some features associated with a positive effect of tDCS and tRNS, in comparison with some of the previous studies using different paradigms and reporting a significant effect of stimulation [9], [16], [17]. For instance, it has been reported that cathodal tDCS while learning a sequence of finger pressings cued in a screen, slowed learning and anodal tDCS had the opposite effect [38]. However, the outcome measure of such an explicit motor task is reaction time whereas the task we used requires a more complex integration of visual and motor information. Also, based on speed accuracy trade-off, an improvement of skill acquisition in a sequential visual isometric pinch task was observed due to 20 min of anodal tDCS [39]. Thus, it is possible that the outcome measure of the task we used is less sensible to potential effects of stimulation. A straightforward comparison of our behavioural results with previously reported data is prevented by differences in study design which can be crucial for the outcome of an experiment in terms of both brain excitability and behavioural measures [16], [19], [38].

(2) Another limitation derives from the fact that not all the groups showed similar initial performance levels. Analysis of the non-normalized data (Table S2) shows that the cathodal tDCS and hf-tRNS groups were initially better and improved more than the other groups. Consequently, we cannot say whether the different group tendencies in learning curve are due to stimulation or initial performance. Also, our design does not allow for separation of learning-specific effects, and it would have been useful to include blocks with a simple motor task.

(3) We have also to consider that another electrode montage might have been more effective. A previous study investigating visuomotor coordination task with tDCS reported no effect of cathodal stimulation on learning whereas anodal stimulation sped learning both with a M1 or V5-Cz montage [18]. However, only V5-Cz montage was effective in modulation of performance after stabilization; cathodal tDCS improved and anodal tDCS worsened performance [9]. Thus, it is possible that also in our paradigm a V5-Cz montage would prove more effective in modulation of learning or performance.

In summary, we have studied the differential effects of tDCS and tRNS on performance of a visuomotor task and related changes in brain activity. Cathodal tDCS and hf-tRNS showed a tendency to improve learning and lf-tRNS had the opposite effect. Further studies are required to clarify the mechanism by which tRNS influences brain activity and its frequency specificity. Stimulation related changes were found in regions related to task, indicating that the effect of the stimulation in not limited to the location where it is applied but instead is determined by the task being performed. Therefore, future studies should investigate whether stimulating other relevant regions such as the DLPFC and V5 would differentially modulate activity and performance according to their specific role in learning and visuomotor integration. Transcranial stimulation techniques are frequently used as a tool in research of visual and motor processes integration in both healthy and pathological conditions. Nevertheless, as our results suggest protocols should be tested and optimized before clinical application.

## Supporting Information

Figure S1 **Voxels showing significant effect of stimulation condition on the mean BOLD signal.** Mean EPI images were averaged across runs for each subject and then non-parametric statistics were performed to test for systematic differences caused by stimulation. Shown are the results of an F-test with voxelwise thresholding at significance level p = 0.05.
(TIF)

Figure S2 **Areas of increased motor task-related brain activity.** First-level analysis of activity associated with the movement blocks were averaged across runs and conditions. Group statistical images were thresholded with clusters determined by Z>2.3 and a significance threshold of p = 0.05 with cluster correction. Numbers indicate MNI standard space z coordinate. See Table 1 in mains text for list of regions and corresponding peak voxel intensity and coordinates.
(TIF)

Figure S3 **Areas of increased performance-related activity.** First-level analysis of activity associated with the tracking error were averaged across runs and conditions. Group statistical images were thresholded with clusters determined by Z>2.3 and a significance threshold of p = 0.05 with cluster correction. Numbers indicate MNI standard space z coordinate. See Table 2 in mains text for list of regions and corresponding peak voxel intensity and coordinates.
(TIF)

Table S1 **Post-hoc t-tests between consecutive blocks of the first run.**
(DOCX)

Table S2 **Statistical analysis of non-normalized behavioural data.**
(DOCX)
