# Supplementary figures and images for: High-Frequency TRNS Reduces BOLD Activity during Visuomotor Learning
Source: PLoS One. 2013 Mar 20;8(3):e59669. doi: 10.1371/journal.pone.0059669 (PMC3603861; doi:10.1371/journal.pone.0059669)

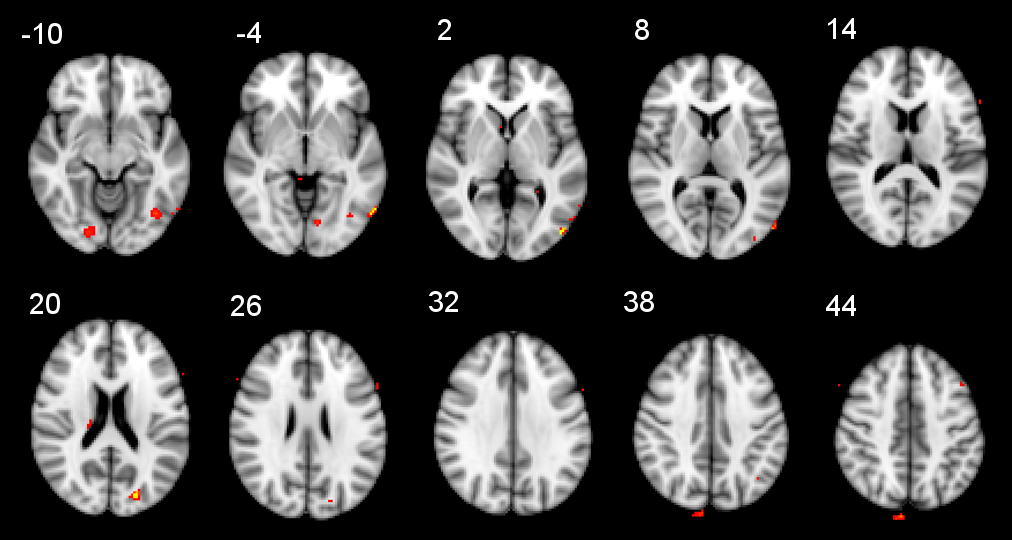

Supplement: Figure S1 — Voxels showing significant effect of stimulation condition on the mean BOLD signal. Mean EPI images were averaged across runs for each subject and then non-parametric statistics were performed to test for systematic differences caused by stimulation. Shown are the results of an F-test with voxelwise thresholding at significance level p = 0.05. (TIF) [file pone.0059669.s001.tif]

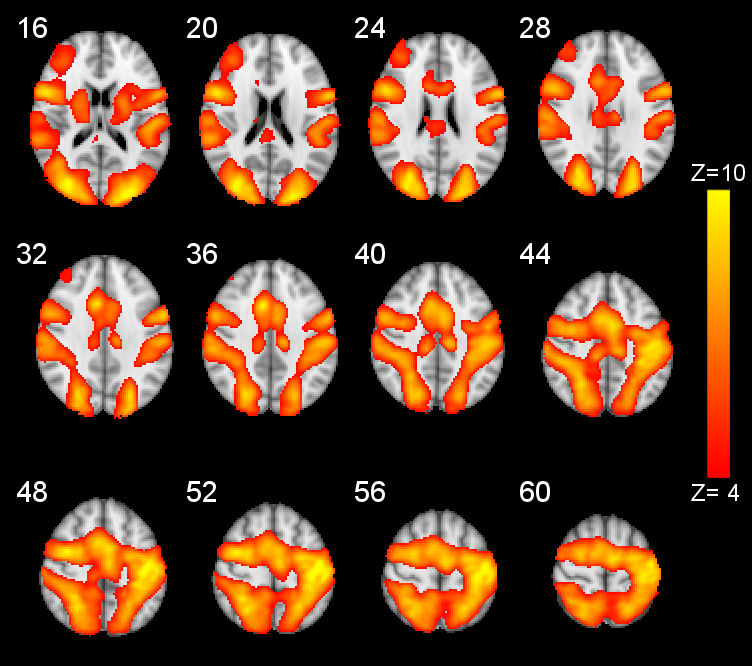

Supplement: Figure S2 — Areas of increased motor task-related brain activity. First-level analysis of activity associated with the movement blocks were averaged across runs and conditions. Group statistical images were thresholded with clusters determined by Z>2.3 and a significance threshold of p = 0.05 with cluster correction. Numbers indicate MNI standard space z coordinate. See Table 1 in mains text for list of regions and corresponding peak voxel intensity and coordinates. (TIF) [file pone.0059669.s002.tif]

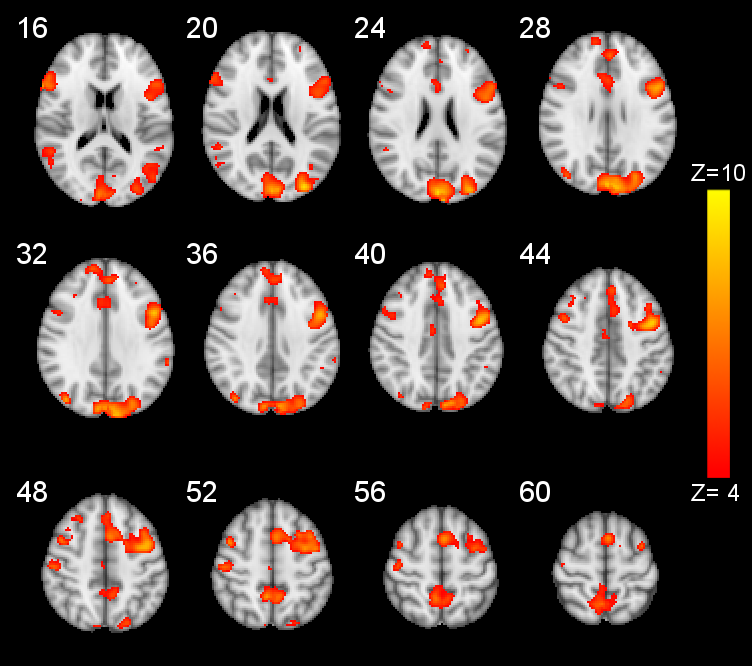

Supplement: Figure S3 — Areas of increased performance-related activity. First-level analysis of activity associated with the tracking error were averaged across runs and conditions. Group statistical images were thresholded with clusters determined by Z>2.3 and a significance threshold of p = 0.05 with cluster correction. Numbers indicate MNI standard space z coordinate. See Table 2 in mains text for list of regions and corresponding peak voxel intensity and coordinates. (TIF) [file pone.0059669.s003.tif]
