# Supplementary material for: High-Frequency TRNS Reduces BOLD Activity during Visuomotor Learning
Source: PLoS One. 2013 Mar 20;8(3):e59669. doi: 10.1371/journal.pone.0059669 (PMC3603861; doi:10.1371/journal.pone.0059669)
Supplement: Table S1 — Post-hoc t-tests between consecutive blocks of the first run. (DOCX) [file pone.0059669.s004.docx]

**Table S1.** **Post-hoc t-tests between consecutive blocks of the first run.**

|  | sham | | anodal tDCS | | cathodal tDCS | | hf-tRNS | | lf-tRNS | |
| --- | --- | --- | --- | --- | --- | --- | --- | --- | --- | --- |
|  | t | p | t | p | t | p | t | p | t | p |
| Blocks 1 - 2 | 3,825 | 0,004* | 4,020 | 0,003* | 8,821 | 0,000* | 3,990 | 0,003* | 4,815 | 0,001* |
| Blocks 2 – 3 | 2,799 | 0,021* | 3,617 | 0,006* | 1,308 | 0,223 | 2,088 | 0,066 | 4,485 | 0,002* |
| Blocks 3 – 4 | 1,050 | 0,321 | 2,321 | 0,045* | 4,319 | 0,002* | 2,370 | 0,042* | 1,248 | 0,243 |
| Blocks 4 – 5 | 1,904 | 0,089 | 1,217 | 0,255 | 1,228 | 0,251 | 2,774 | 0,022* | -0,292 | 0,777 |
| Blocks 5 – 6 | 0,462 | 0,655 | 0,170 | 0,868 | -0,228 | 0,825 | -0,414 | 0,689 | -0,030 | 0,977 |
| Blocks 6 – 7 | -0,099 | 0,924 | 0,305 | 0,767 | 0,832 | 0,427 | 1,147 | 0,281 | 1,250 | 0,243 |
| Blocks 7 – 8 | 1,835 | 0,100 | 1,029 | 0,330 | -0,379 | 0,714 | -0,132 | 0,898 | 0,498 | 0,630 |
| Blocks 8 – 9 | -0,109 | 0,915 | 0,027 | 0,979 | 0,482 | 0,641 | 0,957 | 0,363 | -0,969 | 0,358 |
| Blocks 9 - 10 | 0,645 | 0,535 | 0,867 | 0,408 | 0,200 | 0,846 | -1,492 | 0,170 | -0,498 | 0,630 |

Performance stabilized after at the third block for sham and lf-tRNS and after the fourth for hf-tRNS, anodal and cathodal tDCS.
