# Supplementary material for: High-Frequency TRNS Reduces BOLD Activity during Visuomotor Learning
Source: PLoS One. 2013 Mar 20;8(3):e59669. doi: 10.1371/journal.pone.0059669 (PMC3603861; doi:10.1371/journal.pone.0059669)
Supplement: Table S2 — Statistical analysis of non-normalized behavioural data. (DOCX) [file pone.0059669.s005.docx]

**Table S2. Statistical analysis of non-normalized behavioural data.**

| ANOVA BLOCK * STIMULATION | |
| --- | --- |
| block | F = 71,025; p < 0,001 |
| stimulation | F = 3,566; p =,013 |
| block*stimulation | F = 0,833; p = 0,897 |
| ANOVA RUN * STIMULATION | |
| run | F = 96,975; p < 0,001 |
| stimulation | F = 4,024; p = 0,007 |
| run*stimulation | F = 0,469; p = 0,875 |

A 30 (block) x 5 (stimulation condition) repeated measures ANOVA revealed a significant effect of block as well as stimulation. The same is observed when analyzing the averaged scores per run with a 3 (run) x 5 (stimulation condition) repeated measures ANOVA. This is caused by the better initial performance of cathodal and hf-tRNS groups.
